# Amyand's Hernia: Rare Presentation of a Common Ailment

**DOI:** 10.1155/2015/629127

**Published:** 2015-10-20

**Authors:** Sanjeev Singhal, Anu Singhal, Sanjay Singh Negi, Rahul Tugnait, Pankaj Kumar Arora, Bishwanath Tiwari, Pawan Malik, Lav Gupta, Amit Bimal, Abhishek Gupta, Rahul Gupta, Pushkar Chouhan, ChandraKant Singh

**Affiliations:** ^1^Department of Surgery, Northern Railway Central Hospital, Basant Lane, New Delhi 110055, India; ^2^Department of Radiology, ESI PGIMSR, Basaidarapur, Ring Road, New Delhi 110015, India; ^3^GI and HPB Surgery, Dr. BL Kapoor Memorial Hospital, Pusa Road, New Delhi 110005, India

## Abstract

Inguinal hernia with vermiform appendix as content is known as Amyand's hernia. It is a rare entity but we encountered four cases within six months. A 52-year-old female had high grade fever and evidence of inflammatory pathology involving the ileocaecal region. She was initially managed conservatively and subsequently underwent exploratory laparatomy. The appendix was perforated and herniating in the inguinal canal. Appendectomy was done with herniorrhaphy without mesh placement. A 74-year-old male with bilateral inguinal hernia, of which, the right side was more symptomatic, underwent open exploration. Operative findings revealed a lipoma of the sac and a normal appearing appendix as content. Contents were reduced without appendectomy and mesh hernioplasty was performed. A 63-year-old male with an obstructed right sided hernia underwent emergency inguinal exploration which revealed edematous caecum and appendix as content without any inflammation. Contents were reduced without any resection. Herniorrhaphy was performed without mesh placement. A 66-year-old male with an uncomplicated right inguinal hernia underwent elective surgery. The sac revealed an appendix with adhesions at the neck. Contents were reduced after adhesiolysis and hernioplasty was performed with mesh placement. Emphasis is made to the rarity of disease, variation in presentation, and difference in treatment modalities depending upon the state of appendix.

## 1. Introduction

Various organs have been described as contents of inguinal hernia. Of these, one of the rarest is the vermiform appendix. The incidence of having a normal appendix within the inguinal hernial sac varies from 0.5% to 1%. Appendicitis in the inguinal canal occurs even less frequently, with the reported incidence being between 0.07% and 0.13%. However, incidence as high as 1% has also been reported. Perforated appendix and periappendicular abscess formation within an inguinal hernia sac is even rarer. The incidence of appendicitis in general population is about 8% but only around 0.1% of all cases of appendicitis present in an inguinal hernia [1–4]. The term Amyand's hernia has been used to describe varying clinical scenarios like (a) the occurrence of an inflamed appendix within an inguinal hernia (b), finding a perforated appendix within an inguinal hernia (c), or the presence of a noninflamed appendix within an irreducible inguinal hernia [5]. This anomaly was first described by Amyand in 1735. The eponym Amyand's hernia was first coined by Creese in 1953 and then by Hiatt and Hiatt in 1988, followed by Hutchinson in 1993 [1].

We encountered 4 cases of this rare disease over a relatively short period of about 6 months. This prompted us to present our series of cases along with a detailed review of literature.

## 2. Case 1

**Figure 1:**
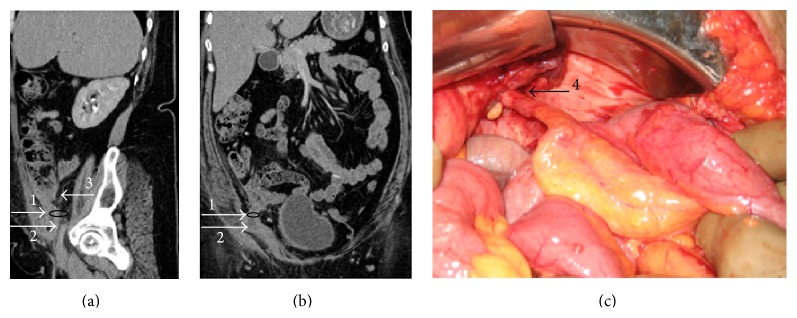
(a) and (b) display the sagittal and coronal views, respectively, of CECT of the first case: 1, black ring depicts the region of right deep ring defect; 2 depicts the herniating appendix; and 3 denotes the caecum. (c) is an operative photograph of the first case showing 4, the inflamed appendix from caecum to its entry in the inguinal canal.

A 52-year-old lady presented with discomfort in right inguinal region. Ultrasound (USG) revealed a small collection in the inguinal canal which was aspirated. She was treated conservatively with antibiotics. After 2 weeks, she again presented with high grade fever, pain in the right iliac fossa and inguinal area, and a lump in the right iliac fossa. Computed tomography (CT) revealed a mass in right iliac fossa where appendix could not be delineated (Figures 1(a) and 1(b)). She was worked up for possible appendicitis or abdominal Koch's with infection tracking along ilioinguinal nerve, but all investigations were noncontributory. Her symptoms did not subside even after 6 weeks of antibiotic therapy and a repeat CT was again indicative of inflammatory pathology in the ileocaecal area. She underwent exploratory laparotomy with lower midline incision. The operative findings included perforated appendix herniating into the right inguinal canal (Figure 1(c)) with periappendicular abscess formation and an umbilical hernia. Appendectomy with drainage of pus, lavage of peritoneal cavity, and primary closure of the hernial defects in the inguinal and umbilical region were done. Postoperatively she developed a small collection in the surgical site which was aspirated under USG guidance. She has recurrence of the umbilical hernia but has otherwise remained comfortable over last 8 months of follow-up.

## 3. Case 2

**Figure 2:**
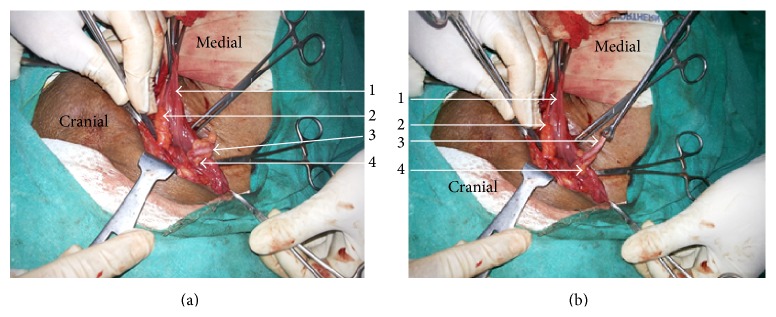
(a) and (b) are operative photographs of the second case showing 1, the opened right indirect hernial sac; 2, lipoma of the sac wall; 3, normal healthy appendix as a content of the sac; and 4, a normal healthy mesoappendix.

A 74-year-old gentleman presented with swellings in both inguinal areas, right more than left, which were gradually progressive over the previous 6 months. The swellings increased on standing and walking but the right sided swelling was not completely reducible. He was a known case of hypertension and coronary artery disease and had undergone bypass surgery 13 years back. A diagnosis of bilateral indirect inguinal hernia, right more than left, was made. He underwent unilateral open inguinal hernia surgery under local anaesthetic block, because of poor cardiac status. Right side was operated as it was partially irreducible and more symptomatic. Operative findings included a lipoma of the sac wall and a normal looking appendix as content of the indirect hernial sac (Figures 2(a) and 2(b)). The adhesions at the neck of the sac were separated and the contents reduced. Mesh hernioplasty was performed using a composite mesh of polypropylene and poliglecaprone. Patient had an uneventful postoperative recovery and is maintaining good health on 6-month follow-up.

## 4. Case 3

A 63-year-old gentleman presented with a longstanding right sided inguinal hernia which had become irreducible for 1 day. The patient had associated history of nonpassage of stools and flatus along with multiple episodes of bilious projectile vomiting over the same duration. He had tachycardia, and on examination there was an irreducible right sided inguinal hernia with tenderness and erythema of the overlying skin. A provisional diagnosis of obstructed right sided inguinal hernia with impending strangulation was made. Patient was taken up for emergency surgery.

**Figure 3:**
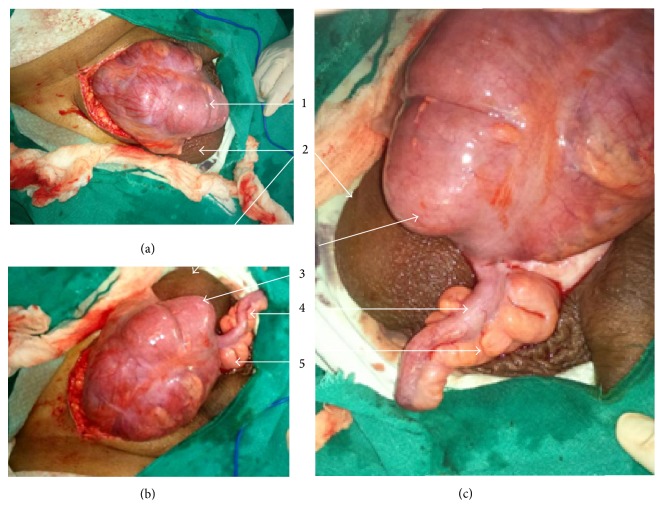
(a), (b), and (c) are operative photographs of the third case showing 1, the obstructed right sided hernial sac; 2, scrotum; 3, caecum; 4, appendix; and 5, mesoappendix. The caecum, appendix, and mesoappendix are edematous but otherwise healthy and show no signs of inflammation.

Intraoperatively, an obstructed indirect sac was found with the level of obstruction at deep ring. It had caecum and appendix as contents. The bowel, though oedematous, was viable and healthy (Figures 3(a), 3(b), and 3(c)). The obstruction was relieved, contents were reduced, and herniorrhaphy (Bassini's repair) was performed. The patient had an uneventful recovery and is in good health on a 5-month follow-up.

## 5. Case 4

A 66-year-old male patient presented with history of left inguinal swelling for 6 months which was reducible and used to increase in size on straining. After thorough examination it was diagnosed as left indirect uncomplicated inguinal hernia. Patient had no significant comorbidities. He was taken up for surgery after all necessary investigations and preanaesthetic fitness.

**Figure 4:**
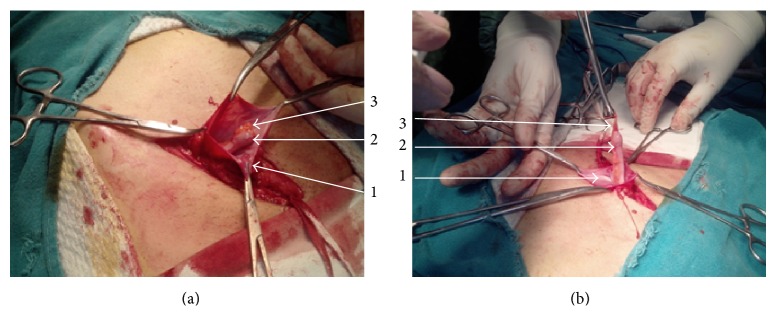
(a) and (b) are operative photographs of the fourth case showing 1, the opened right indirect hernial sac; 2, normal healthy appendix as a content of the sac; and 3, a normal healthy mesoappendix.

He underwent hernioplasty under inguinal block. Intraoperatively an indirect sac was found and dissected free of cord structures. On opening, it revealed appendix as content. There were flimsy adhesions of mesoappendix with the neck of the sac (Figures 4(a) and 4(b)). The adhesions were gently broken and appendix was reduced. Hernioplasty was completed using a composite mesh of polypropylene and poliglecaprone. Patient had an uneventful postoperative recovery and has remained comfortable over last 3 months of follow-up.

## 6. Discussion

Amyand's hernia, which means the presence of a normal or pathological appendix as a content of inguinal hernia, is a rare clinical entity. D'Alia et al. reported an incidence of just 0.08%, in 1341 patients of inguinal hernias over a 13-year period in Messina University Hospital in 2003 [7]. Presence of appendicitis in inguinal hernia is even more uncommon. Solecki et al. observed that acute appendicitis was found in 0.62% of all groin hernia sacs [8] whereas Ryan reported that only 11 of 8,692 cases of acute appendicitis occurred in external hernia of all forms [9]. The incidence of acute appendicitis occurring in a hernial sac varies between the 0.13% reported by Ryan in 1937 and the 1% reported by Carey in 1967 [10]. Thomas et al. reported only 7 cases of acute appendicitis occurring in an external hernia sac during 8 years [11]. In a review of 18 cases, the median age was 42 years, with the oldest age noted in literature being 89 years. However, age of these cases ranges from 3 weeks to 88 years [12]. It is more common in men and almost always on the right side probably because of the normal anatomical position of the appendix and also because right sided inguinal herniae are more common. Left sided Amyand's herniae are even rarer. They may be associated with situs inversus, malrotation, or a very mobile caecum [2]. Though all our cases were right sided, one was in a female. All our male patients were over 60 years and the female patient was 52 years old. The average age of our patients was 63.75 years.

Appendix in a hernial sac was first reported in a femoral hernia, in 1731 by De Garengeot [13]. Appendix in an inguinal hernia was first reported by Amyand, sergeant surgeon to King George II of England, on December 6, 1735. His patient was Hanvil Anderson, an 11-year-old boy, with an appendiceal abscess, presenting with “a fistula between the scrotum and thigh.” He treated the child by a transhernial appendectomy and pathology revealed a chronically inflamed appendix included within the inguinal hernia sac and perforated by a previously swallowed pin. He not only described the first case of appendicitis in the inguinal canal (later named as Amyand's hernia), but also performed the world's first recorded successful appendectomy. The patient eventually recovered and was “discharged with a truss, which he was ordered to wear for some time.” The case was published in the Philosophical Transactions of the Royal Society of London. Though the appendectomy was successful, the hernia later recurred [4, 13–15]. Therefore, in his honour, an inguinal hernia containing the vermiform appendix, whether it is inflamed or not, is called “Amyand's hernia.” Interestingly the first appendicectomy performed in USA by Robert J Hall a century and a half later, in 1886, was also performed in a case of perforated appendix within an inguinal hernia, another example of Amyand's hernia [16].

The pathophysiology of appendicitis in Amyand's hernia is unknown. Majority of the cases have appendicitis incarcerated in a hernia. Weber et al. proposed that, due to herniation, the appendix becomes more vulnerable to microtrauma causing inflammation and adherence of appendix to the hernial sac due to fibrosis. This hypothesis that inflammatory swelling may lead to incarceration and subsequent impaired blood supply and bacterial overgrowth was supported by several other authors. Muscle contraction and changes in abdominal pressure cause compression of appendix resulting in decreased blood supply and secondary inflammation [1, 2]. It is difficult to determine whether appendicitis is the main pathology or if the primary event is strangulation of the herniated appendix, leading to ischemic necrosis and secondary inflammation.

Preoperative diagnosis requires high index of clinical suspicion as there are no typical or pathognomonic symptoms. Clinical picture may vary depending on the extent of periappendicular inflammation and the presence or absence of peritoneal contamination. It is a differential diagnosis of an irreducible inguinal hernia and inguinal lymphadenitis as well as an acute scrotum. It may present as a strangulated hernia, testicular torsion or tumors, epididymitis, a fistula or an abscess of the scrotum, thigh, or abdominal wall, or an incidental finding. Tenderness over McBurney's point is usually absent [5, 17]. Due to these facts, it is difficult to reach a clinical diagnosis of Amyand's hernia. Diagnosis is usually made intraoperatively when the patient undergoes surgical exploration. In one review of 60 cases over a 12-year period, only one case was diagnosed preoperatively [18]. Laermans et al. in 2003 reported only two cases where diagnosis was established prior to surgery. Ultrasound often demonstrates a potentially inflammatory mass within the hernial sac. An abdominal CT scan could diagnose the condition but is usually not performed [5, 17]. Diagnosis is made by demonstration of an inguinal herniation containing a blind-ending tubular structure with thickened walls, in connection with the caecum. Multiplanar reconstructions are most useful in order to better visualize the appendix and demonstrate its relationship with surrounding structures [17]. The CT scan done in our first case could only pick up the abnormality retrospectively.

There are two issues in treatment of Amyand's hernia, whether to perform an appendectomy or not and whether or not to use mesh in hernia repair. The presence or absence of inflammation of appendix is an important determinant of appropriate treatment. If appendicitis is present, a transherniotomy appendectomy is advocated. Associated intra-abdominal abscess should be drained either percutaneously or by open drainage. In presence of pus or perforation of the organ, mesh repair is contraindicated due to increased risk of complications. A normal looking appendix in the hernial sac does not always require appendectomy. Appendectomy adds the risk of infection to an otherwise clean procedure. Superficial wound infection increases morbidity; and deep infection may contribute to hernia recurrence [14]. However, Ofili reported two patients who developed acute appendicitis after repair of inguinal herniae which contained the appendix, along with 11 patients who had appendicectomies at the time of herniorrhaphy, without developing any wound infections or hernia recurrences. He, therefore, advocated “incidental” appendicectomies [19]. Whether to remove or leave behind a normal appendix is a clinical dilemma because no evidence-based information exists. The decision should be based on common sense, taking into account the patient's age, life expectancy, life-long risk of developing acute appendicitis, and the size and overall anatomy of the appendix. Also, in cases where there is no inflammation, and appendix has merely been reduced, there is no contraindication to using a mesh [14], though some authorities recommend that the use of synthetic mesh should be avoided even if the appendix appears normal, as subclinical contamination is bound to occur [2].

A scheme of classification and management has been given by Losanoff and Basson [6] (Table 1).

We conclude that the presence of the appendix in an inguinal hernial sac, referred to as “Amyand's hernia,” is a rare entity and appendicitis within the hernial sac is even less frequently encountered. Preoperative diagnosis is difficult and the condition is usually diagnosed intraoperatively. Surgeons should be aware of the condition and the recommended line of management, so that they are able to offer appropriate treatment.

## Figures and Tables

**Table 1:** Classification of Amyand's hernias and their plan of management [6].

Classification	Description	Surgical management
Type I	Normal appendix in inguinal hernia	Hernia reduction; mesh repair; appendectomy in young patients
Type II	Acute appendicitis within an inguinal hernia and no abdominal sepsis	Appendectomy through hernia; primary repair of hernia; no mesh
Type III	Acute appendicitis within an inguinal hernia or the abdominal wall or peritoneal sepsis	Laparotomy; appendectomy; primary repair of hernia; no mesh
Type IV	Acute appendicitis within an inguinal hernia with related or unrelated abdominal pathology	Management as detailed above for hernia type I–III and treat the second pathology as appropriate
